# Comprehensive Health Assessment for Children in Out-of-Home Care: An Exploratory Study of Service Needs and Mental Health in a Norwegian Population

**DOI:** 10.1007/s10578-023-01619-5

**Published:** 2023-10-12

**Authors:** Monica Haune, Alexander Nissen, Øivin Christiansen, Trine M. Myrvold, Torleif Ruud, Einar R. Heiervang

**Affiliations:** 1 Institute of Clinical Medicine, University of Oslo, Blindern, P.O box 1171, Oslo, 0318 Norway; 2 Division for Forced Migration and Refugee Health, Norwegian Centre for Violence and Traumatic Stress Studies, Oslo, Norway; 3 Regional Centre for Child and Youth Mental Health and Child Welfare, NORCE Norwegian Research Centre, Bergen, Norway; 4 Norwegian Institute for Urban and Regional Research, Oslo Metropolitan University, Oslo, Norway; 5 Mental Health Services, Akershus University Hospital, Lørenskog, Norway; 6 Oslo University Hospital, Oslo, Norway; 7 Innlandet Hospital Trust, Innlandet, Norway

**Keywords:** Children in out-of-home care, Comprehensive assessment, Service needs, Mental health

## Abstract

A comprehensive model for routine multi-disciplinary health assessment for children in out-of-home care was piloted in a Norwegian region. This paper reports on identified service needs and mental disorders among 196 children (0–17 years) receiving the assessment. Cross-sectional data was extracted from assessment reports. Results show needs across a range of services, with a mean of 2.8 recommended services for children aged 0–6 and 3.3 for children aged 7–17. Mental disorders were identified in 50% of younger children, and 70% of older children. For all children, overall service need was associated with mental disorders, in addition to male gender among younger children. Need for specialized mental health services was associated with mental disorders among younger children and increasing age among older children. The high frequency of service needs and mental disorders illustrate the importance of offering comprehensive health assessments routinely to this high-risk child population and necessitates coordinated service delivery.

**Supplementary Information:**

The online version contains supplementary material available at 10.1007/s10578-023-01619-5.

## Introduction

Children who are removed from their family of origin by the child welfare system and placed in out-of-home care, also referred to as looked-after children or children in care, often present with extensive and undetected mental and physical health care needs [1–4]. The rates of health problems are significantly higher than that of other child populations, regardless of socioeconomic background [5]. These problems are hypothesized to stem from interactions between prenatal factors, genotype, exposure to maltreatment, emotional deprivation and attachment disruption [6]. Exposure to multiple adverse childhood experiences has been convincingly documented to increase the risk for poor mental and physical health, and this risk extends into adulthood [7–9]. In the current study we use the term out-of-home care (OOHC) to denote children living in long-and short term foster care, kinship care or residential care.

A systematic review and meta-analysis of mental disorders among children in the Child Welfare Services (CWS), reported a pooled prevalence of 49%, with disruptive disorders, anxiety disorders, and depressive disorders, occurring most frequently [10]. Studies of mental health in children shortly after entering care, have reported higher prevalence rates, specifically for trauma and adjustment related disorders (30–40%) [11, 12]. Furthermore, children in OOHC have an increased risk of attachment difficulties, such as reactive attachment disorder [13], which has been associated with complex neuropsychiatric problems and a range of comorbid disorders [14, 15]. The variation in prevalence rates across studies are assumed to reflect differences in study design, rather than actual differences in the studied populations [16]. In addition to extensive mental health problems, children in OOHC have poorer physical health [17], a higher prevalence of developmental difficulties [5], and lower educational attainment [18] than their non-fostered peers.

A stable foster home is essential, but often insufficient to ensure healthy mental and physical development [19]. Previous studies have shown an over-reliance on CWS workers’ and foster carers’ ability to identify needs, especially needs concerning mental health and development [20–22]. Decades of research, and international best practice guidelines, recommend routine comprehensive health assessments shortly after entry into OOHC, using standardized measures, with the purpose of identifying needs, providing early interventions, and making appropriate referrals [2, 5, 23–30]. Current assessment practices vary across countries and jurisdictions, with regards to what is covered by the assessment, type of measures used, type and number of professionals involved, and often there is no health assessment offered at all [17, 31–33]. A physical evaluation performed by a pediatrician is more common than comprehensive assessments of physical (including dental), mental and developmental health performed by a multi-disciplinary team [5]. Assessment models often lack a systemic understanding of this specific population and sufficient consideration of the importance of the child-carer relationship [34]. Examples of comprehensive health assessment models, with specialized clinicians using a systematic, ecological and therapeutic approach, are described in the studies by Chambers and colleagues from the Alternate Care Clinic [2] and Milburn and colleagues with the Stargate Early Intervention Programme [12].

Despite international recommendations, many children entering OOHC do not receive a health assessment [2], even in face of severe health problems [35]. Studies of service use among children in OOHC report that they are often not receiving services relative to their needs [32, 36–38]. They are especially underserved by child and adolescent mental health services (CAMHS) [39–41]. Severity of emotional and behavioral problems have been associated with increased service use in general, and with the use of mental health services specifically [13, 36, 38, 42]. Some studies report that behavioral disorders, but not emotional disorders, predict service use among children in OOHC [43, 44]. Others have found no differences between types of mental health problems in this respect [13, 36, 45]. Maltreatment history has been associated with mental health services use, with higher service use among children exposed to sexual or physical abuse than among children exposed to neglect, even after controlling for the severity of symptoms [38, 42, 46]. Older age has been associated with poorer mental health and increased use of mental health services, [38, 42], but this may be confounded by age at entry into OOHC [47, 48]. Studies of the effect of gender on service use in this child population are inconclusive, some reporting higher service use among boys [38, 49] while others report no gender differences [42, 43, 46]. Other factors assumed to contribute to children in OOHC being underserved by CAMHS include the service’s lack of availability and commonly applied intake criteria requiring the child to live in a stable home environment to receive treatment [13, 50, 51].

In Norway, the 356 municipalities have the main responsibility for the CWS, primary health services, kindergartens and primary and secondary schools, while specialized health care services are organized and funded on a state level. CAMHS is responsible for the assessment and treatment of mental disorders, and requires referral from GP, other specialized health care services or Child Welfare Services. As in the other Nordic countries, health care is free for all children, and children in OOHC are served by the same health care services as the general child population. Comprehesive health assessments have not previously been provided to children entering OOHC in Norway, nor are there any national figures regarding service use for this child population [51]. A study of Norwegian school aged children in foster care, reported that 21.9% were currently in contact with CAMHS [52]. An official report concerning the health care of Norwegian children in OOHC concluded that their mental health needs were often neglected, and called for a comprehensive health assessment for all children after placement. The Children at Risk Evaluation (CARE) assessment was developed and piloted as a response to this, as the first of its kind in Norway [51]. The model applied a trauma informed and ecological understanding of the complex symptomatology these children present with, in line with recent recommendations [34, 53], with the purpose identifying mental, physical, and developmental needs, and provide recommendations for services and foster carer supervision. The current study was as part of a larger evaluation of the CARE assessment model, contributing to the development of a national assessment model.

Our main aims were to describe the service needs identified through the CARE assessment model and present the prevalence of mental disorders in the sample. In addition, we aimed to explore whether age, gender, maltreatment type and mental disorders were associated with the need for specialized mental health services and with service need in general.

## Methods

### Sample Recruitment

The CARE assessment model was piloted in the southern region of the Norwegian state CWS (Regional Office for Children, Youth and Family Affairs for Southern Norway), covering 5 counties and 83 municipalities, with a total population of about 1 million inhabitants. Municipal CWS offices from this region were invited to refer all children between the ages of 0 to 17 years who entered OOHC during the 2 year inclusion period of the study. Children aged 0–6 years, and 7–17 years were referred to two different specialized assessment teams. The team for 0–6-year-olds was established at a Child and Family Center run by the CWS, while the team for 7–17-year-olds was established at a county CAMHS department. Based on the time and staff available to this pilot study, and the number expected to enter OOHC in this specific region, assessment of 100 children over a 2-year period at each site was considered a realistic target. The CWS offices within the region were informed about the study at meetings before and during the study period, and by e-mail.

### The CARE Assessment Model

The two assessment teams consisted of experienced clinicians, specialized in assessment of children within their respective age group. Both teams included child and adolescent psychiatrists, clinical child psychologists, clinical child welfare workers, and an administrative coordinator. Following reception of referral, a date for the assessment day was communicated to the referring CWS office and the carer of the child. The carer accompanied the child to the assessment, where all direct examination of the child was conducted in a single day. Prior to this day, the teams collected information from the CWS, from the carer, and from kindergarten or school personnel about the health and development of the child. From the age of 11 years, the children were invited to complete an online self-report regarding their health and well-being in advance of the assessment day. The assessment covered physical health, mental health, cognitive and social development, attachment, and trauma exposure and symptoms using age appropriate standardized measures. Measures were adapted to the age and developmental level of the child, as well as to the child’s capacity on the assessment day (for detailed description of the applied measures please see supplementary table). Clinical interviews with children and carers included assessment of protective factors and risk factors, relationships, friendships, school situations and interests along with risk behaviors. Access to same-day dental examination was available. Breaks for meals, rest and play were offered throughout the day. At the end of the assessment day, preliminary information regarding assessment results was offered to carer and school aged children, and the team obtained feedback from child and carer regarding their experiences with participation in the assessment. Results on the different measures and examinations were structured and discussed by the team before synthesizing it into a written report sent to the referring CWS office, usually within 2–4 weeks. The reports were structured as follows: family background, CWS history, reasons for placement, developmental history, medical history, health and care needs according to diagnostic classifications, recommendations for supervision of foster carers and for referrals to services. The child’s CWS caseworker was responsible for following up on the recommendations. In some instances, immediate service contact was deemed necessary, and the clinicians themselves initiated referrals.

### Data Extraction

Data were extracted and coded from 196 assessment reports by the first, third and fourth authors and plotted into a retrospective form. Final coding was determined by consensus or in consultation with the senior investigator (last author) when deemed necessary.

### Ethical Procedures

The Norwegian Centre for Research Data approved of the study. The Norwegian Directorate for Children, Youth and Family Affairs and the Council for Confidentiality and Research provided the study exemption from confidentiality for CWS case-workers, mandating the CWS office to consent to the child’s participation in the health assessment in place of biological parents. The CWS caseworkers provided age-appropriate information about the assessment to the children.

### Measures

The measure of demographic aspects, types of maltreatment, types of mental disorders, and service recommendations were based on information derived from the assessment reports written by the teams. They will be presented in Tables 1, 2, 3, 4, 5 and 6.

### Demographic Information

Age was obtained from national identity numbers and treated as a continuous variable in regression analyses, and as a categorical variable in bivariate analyses, with four age groups (0–2, 3–6, 7–11, and 12–17). Gender was measured as a dichotomous variable.

### Maltreatment Type

Based on previous studies reporting increased service use among children exposed to sexual and physical abuse [42, 46] we compared children with and without reported exposure to physical or sexual abuse. Based on the reported reasons for placement, a dichotomized variable was created.

### Mental Disorders

The diagnostic classification used by Norwegian specialist health services is the ICD-10 [54]. This was used in the assessment, for classifying mental disorders in children aged 6 years and above. For children aged 0 to 5 years, the clinicians used the DC:0–5; Diagnostic Classification of Mental Health and Developmental Disorders of Infancy and Early Childhood [55]. The diagnostic evaluation conducted by the teams was based on semi-structured interviews and multi-informant measures. For the purpose of the current study, diagnoses were coded as present when it was clearly concluded in the reports that diagnostic criteria were met. For the analyses, the diagnoses were grouped into categories according to the ICD-10. Disorders from the DC:0–5 were translated into the corresponding ICD-10 categories based on crosswalks described in the revised and updated DC:0–5 [56].

### Recommended Services

Recommended services described in the reports included: General practitioner (GP), community health care services (municipal health services for families, children and adolescents, including school health services), dental health services, physiotherapy, psychosocial and educational support in kindergarten and school, educational psychology services (EPS), CAMHS, and specialized somatic care (including habilitation services). Because all children living in OOHC are in contact with the CWS, only services outside of the CWS were coded. For each service, recommendations were entered one of the following four categories: *new referral, continued contact, watchful waiting, or no referral needed.* Watchful waiting was used when the clinician’s recommended referral to service if problems persisted or in the absence of positive development despite stable care.

When analyzing the current service need for CAMHS and for services in general and the influence of explanatory factors, we combined the recommendation categories *new referral* and *continued contact*. Service need in general was modelled as a continuous variable, based on the total number of services recommended.

### Statistical Analyses

All analyses were conducted using STATA 16 (STATA Corporation, College Station, TX, USA). As two different teams, one localized in CWS and one in CAMHS, assessed two different age cohorts, analyses were done separately for children aged 0–6 and 7–17.

To investigate frequencies and distributions of predictors and outcome variables, we used cross-tabulations, for both the whole sample and for the sample split by age group/assessment team. Chi-square tests for equal proportions (or Fisher’s exact test) were used to investigate bivariate associations between key predictors (i.e. age, gender, maltreatment type and mental disorder) and need for CAMHS. Logistic regression analysis was used to evaluate crude and adjusted associations between key predictors and recommendations for CAMHS. In order to avoid unstable parameter estimates, we only included diagnostic categories with five or more children in the outcome category (i.e. ≥ 5 with *current CAMHS needs*). Two-sample, independent t-test was used to evaluate whether the service need in general differed between the two main age groups (0–6 vs. 7–17). Linear regression analysis was used to evaluate crude and adjusted associations between key predictors and service need in general. Unstandardized regression coefficients (*b*) are reported in this analysis. We used an alpha level of 0.05 to indicate statistically significant results. Given the exploratory nature of the study, no formal power analyses was conducted. All comparisons will thus be considered exploratory regardless of the significance level found.

### Missing Data

Two children in the 7–17 age group were missing their precise age and were excluded from the bivariate and the multivariate models.

## Results

### Sample Characteristics

Table 1 describes the demographic and placement characteristics of the children who received the assessment within two calendar years. The sample included 196 children residing in OOHC, of which 46.4% were girls. The sample of younger children had a higher representation of boys (61.7%, *n* = 58) than girls (38.3, *n* = 36). The mean age was 3.5 years in the younger age group and 10.8 years in the older age group. Of the total sample 36% (*n* = 70) had 1–2 registered reasons for placement, whereas 55.3% (*n* = 108) had 3–4 registered reasons for placement, with emotional neglect/abuse, physical abuse and practical/physical neglect occurring most frequently. Children were accepted for the health assessment regardless of time spent in care prior to referral. More than two thirds of the children received the assessment within 6 months after placement. At the time of assessment, around half of the children lived in emergency foster care, awaiting long term foster or residential care. Only seven children lived in residential care, all from the older age group.

**Table 1:** Demographic and placement characteristics for children in out-of-home care (0–17 years) assessed with the CARE assessment model between 2017 and 2020

	Total sample N = 196 % (n)	Ages 0–6 N = 94 % (n)	Ages 7–17 N = 102 % (n)
Mean age (years, SD)	7.3 (4.4)	3.5 (2.0)	10.8. (2.8)
Female	46.4 (91)	38.3 (36)	53.9 (55)
Placement type (n = 186)			
Emergency foster care	53.2 (99)	55.2 (48)	51.5 (51)
Foster home	39.3 (73)	41.4 (36)	37.4 (37)
Residential care	3.8 (7)	0	7.1 (7)
Other	3.8 (7)	3.5 (3)	4.0 (4)
Registered reasons for placement^a^			
Physical abuse	58.7 (115)	56.4 (53)	60.8 (62)
Sexual abuse	6.1 (12)	5.3 (5)	6.9 (7)
Emotional neglect/abuse	77.0 (151)	81.9 (77)	72.6 (74)
Practical/physical neglect	56.1 (110)	66.0 (62)	47.1 (48)
Parent substance abuse	29.1 (57)	30.9 (29)	27.5 (28)
Parent mental health	31.1 (61)	28.7 (27)	33.3 (34)
Other reasons	38.3 (75)	31.9 (30)	44.1 (45)
Maltreatment type^b^			
Physical or sexual abuse	59.7 (117)	57.5 (54)	61.8 (63)
Other maltreatment types^c^	31.1 (61)	35.1 (33)	27.5 (28)
No reported maltreatment	9.2 (18)	7.5 (7)	10.8 (11)
Time from placement to assessment (n = 185)			
0 to 2 months	16.2 (30)	25.0 (22)	8.3 (8)
3 to 6 months	49.2 (91)	58.0 (51)	41.2 (40)
7 to 12 months	14.6 (27)	8.0 (7)	20.6 (20)
> 12 months	20.0 (37)	9.1 (8)	29.9 (29)

^a^Because of multiple reasons, the figures exceed the number of children

^b^Indicator variables coded as physical and/or sexual abuse present: Y/N

^c^Includes children exposed to emotional abuse, emotional neglect or physical/practical neglect with no reported exposure to physical or sexual abuse

### Prevalence of Mental Disorders

The proportion of children meeting criteria for at least one ICD-10 or DC:0–5 disorder was 50.0% in the younger age group and 69.6% in the older age group. Table 2 shows the prevalence of specific disorders, and of categories of disorders according to the ICD-10. All disorders under the category neurotic stress related and somatoform disorders belongs to the ICD-10 subcategory *Reaction to severe stress and adjustment disorders*. These disorders are the most frequently occurring, with particularly high prevalence among older children (58.8%). Among the 118 children with mental disorders, 38 (32.2%) children had more than one disorder, and of these, 16 (34.0%) were in the younger age group and 22 (31.0%) were in the older age group.

**Table 2:** Prevalence of ICD-10 disorders in children (0–17 years) in out-of-home care according to the CARE assessment

ICD-10 disorder	Total sample (N = 196)	Ages 0–6 (N = 94)	Ages 7–17 (N = 102)
	% (n)	% (n)	% (n)
Any disorder	60.2 (118)	50.0 (47)	69.6 (71)
Neurotic, stress-related and somatoform disorders^a^	41.3 (87)	22.3 (22)	58.8 (65)
Post-traumatic stress disorder	19.9 (39)	16.0 (15)	23.5 (24)
Other reactions to severe stress	18.9 (37)	0	36.3 (37)
Adjustment disorders	3.1 (6)	3.2 (3)	2.9 (3)
Specific phobias	0.5 (1)	0	1.0 (1)
Other trauma, stress and/or deprivation disorder of infancy/early childhood^b^	2.0 (4)	4.3 (4)	0
Behavioral and emotional disorders	18.9 (37)	17.0 (16)	20.6 (21)
ADHD	5.1 (10)	2.1 (2)	7.8 (8)
Oppositional defiant disorder	1.0 (2)	1.1 (1)	1.0 (1)
Separation anxiety disorder	0.5 (1)	0	1.0 (1)
Childhood emotional disorder, unspecified	0.5 (1)		1.0 (1)
Reactive attachment disorder	6.1 (12)	6.4 (6)	5.9 (6)
Disinhibited attachment disorder	3.6 (7)	6.4 (6)	1.0 (1)
Transient tic disorder	2.0 (4)	1.0 (1)	2.9 (3)
Vocal and motor tic disorder (Tourette’s)	0.5 (1)	.	1.0 (1)
Nonorganic enuresis/encopresis	3.1 (6)	1.1 (1)	4.9 (5)
Unspecified behavioral and emotional disorders	0.5 (1)	0	1.0 (1)
Mood (affective) disorders	8.2 (16)	16.0 (15)	1.0 (1)
Moderate depressive episode	0.5(1)	0	1.0 (1)
Disorder of dysregulated anger and aggression of early childhood^b^	7.7 (15)	16.0 (15)	0
Disorders of psychological development	5.1 (10)	5.3 (5)	4.9 (5)
Other developmental disorders of speech and language	1.0 (2)	1.1 (1)	1.0 (1)
Developmental disorders of speech and language, unspecified	2.0 (4)	1.1 (1)	2.9 (3)
Mixed disorder of scholastic skills	2.0 (4)	1.1 (1)	2.9 (3)
Other^c^	2.0 (4)	2.1 (2)	2.0 (2)
Eating disorder, unspecified	0.5 (1)	1.1 (1)	0
Overeating associated with other psychological disturbances	1.0 (2)	1.1 (1)	1.0 (1)
Mild mental retardation	0.5 (1)	0	1.0 (1)

^a^All disorders under the category neurotic stress related and somatoform disorders belongs to the ICD-10 subcategory “Reaction to severe stress and adjustment disorders”

^b^From DC:0–5 Diagnostic Classification of Mental Health and Developmental Disorders of Infancy and Early Childhood

^c^Disorders with 5 children or fewer in each category.

### Recommended Services Following Assessment

Among the children aged 0–6 years, 45.7% (*n* = 43) were recommended 1 or 2 services, 27.7% (*n* = 26) were recommended 3 or 4 services, and 11.7% (*n* = 11) were recommended 5 or more services. Among children aged 7–17 years, 27.5% (*n* = 28) were recommended 1 or 2 services, 51.0% (*n* = 52) were recommended 3 or 4 services, and 20.6% (*n* = 21) were recommended 5 or more services. The percentage of children needing no services outside of the CWS was 14.9% (*n* = 14) in the younger age group and 1% (*n* = 1) in the older age group. The mean number of services recommended for children aged 0–6 years was 2.80 (95% CI 1.91–2.65), and for the children aged 7–17 the mean number of services was 3.30 (95% CI 3.00–3.60). The difference in mean number of recommended services between the two age cohorts was highly significant (*p* < .001).

Table 3 presents the services recommended across the recommendation types The most frequent new service recommendations in the younger age group are community health care and EPS, and in the older age group EPS and GP. New CAMHS referral was recommended for 22.3% in the younger group and 31.4% in the older group. For children with existing service contacts, continuation of these were recommended to all. The category watchful waiting was most often used for CAMHS, both for the younger (28.7%) and the older (42.2%) age group.

**Table 3:** Service recommendations for children (0–17 years) in out-of-home care after comprehensive, multidisciplinary health assessment

	New referrals to services, % (n)			Continued service use, % (n)			Watchful waiting, % (n)		
	Total	Ages 0–6	Ages 7–17	Total	Ages 0–6	Ages 7–17	Total	Ages 0–6	Ages 7–17
GP	37.8 (74)	23.4 (22)	51.0 (52)	6.1 (12)	5.3 (5)	6.9 (7)	2.6 (5)	5.3 (5)	0
Community health care^a^	25.0 (49)	33.0 (31)	17.7 (18)	3.6 (7)	4.3 (4)	2.9 (3)	9.2 (18)	1.1 (1)	16.7 (17)
Dental clinic	19.9 (39)	19.2 (18)	20.6 (21)	6.6 (13)	5.3 (5)	7.8 (8)	0.5 (1)	1.1 (1)	0
Physical therapy	9.7 (19)	11.7 (11)	7.8 (8)	1.5 (3)	3.2 (3)	0	6.6 (13)	6.4 (6)	6.9 (7)
CAMHS	27.0 (53)	22.3 (21)	31.4 (32)	6.6 (13)	1.1 (1)	11.8 (12)	35.7 (70)	28.7 (27)	42.2 (43)
Specialized somatic health care services	13.3 (26)	16.0 (15)	10.8 (11)	6.6 (13)	10.6 (10)	2.9 (3)	7.1 (14)	10.6 (10)	3.9 (4)
EPS	43.4 (85)	30.8 (29)	54.9 (56)	8.7 (17)	6.4 (6)	10.8 (11)	15.8 (31)	21.3 (20)	10.8 (11)
Adaptions in school^b^	21.9 (43)	17.0 (16)	26.5 (27)	6.1 (12)	3.2 (3)	8.8 (9)	3.1 (6)	4.3 (4)	2.0 (2)
Other services^c^	25.0 (49)	3.2 (3)	45.1 (46)	0	0	0	1.5 (3)	1.1 (1)	2.0 (2)

^a^Includes municipal health clinics for families, children, and adolescents and school health services

^b^Educational and psychosocial support in kindergarten and school in addition to or without the involvement of Educational Psychology Service

^c^Includes services such as a specific municipal mental health team, optician, or speech therapist

### Associations Between Need for CAMHS and Age, Gender, Maltreatment Type and Mental Disorders

Just below half of the children with a mental disorder were recommended services from CAMHS. The frequency of children being recommended services from CAMHS across age, gender, maltreatment type, and mental disorders is presented in Table 4. In the younger age group, exposure to physical or sexual abuse (*p* = .032), having a mental disorder (*p* < .001), and having a stress-related and adjustment disorder (*p* < .001) was significantly associated with recommendations for CAMHS. Children aged 3 to 6 years were more frequently recommended referral than children aged 0 to 2 years (*p* = .012). In the older age group, no significant associations were found. There is borderline statistical significance for the association between the presence of behavioral and emotional disorders and recommendations for CAMHS (*p* = .051). Table 5 shows the fully adjusted logistic model of CAMHS service needs regressed on predictors. Children in the younger age group with stress-related and adjustment disorders had 23 times higher odds of being recommended services from CAMHS (OR = 23.6, 95% CI [5.65–98.79]). Children in the older age group had 1.2 times higher odds of being recommended services from CAMHS for every 1-year increase in age (OR = 1.20, 95% CI [1.03–1.41]). In the adjusted logistic regression model there were no longer significant associations between reported physical or sexual abuse and recommended services from CAMHS for the younger children.

**Table 4:** Frequency and bivariate test results for recommended services from child and adolescent mental health services (CAMHS)

	Ages 0–6 (*n* = 94)				Ages 7–17 (*n* = 100)			
	N	Recommended services CAMHS % (n)	*p*	ES	N	Recommened services CAMHS % (n)	*p*	ES
Girls	36	19.4 (7)	0.475	0.07	55	43.6 (24)	0.912	− 0.01
Boys	58	25.9 (15)			47	42.6 (20)		
Age categorized within age cohort								
0–2	41	9.8 ( 4)						
3–6	51	**33.3 (17)**	**0.012**	**0.29**				
7–12					68	36.8 ( 25)	0.190	0.18
13–17					31	51.6 (15)		
Maltreatment type								
Physical or sexual abuse	54	**31.5 (17)**	**0.032**	**0.22**	63	41.3 (26)	0.628	− 0.04
Any mental disorder	47	**44.7 (21)**	**< 0.001**	**0.50**	71	47.9 (34)	0.143	0.15
ICD-10 disorders (categorized)								
Neurotic, stress-related, and somatoform disorders	21	**71.4 (15)**	**< 0.001**	**0.61**	60	46.7 (28)	0.390	0.08
Behavioral and emotional disorders	16	37.5 (6)	0.193	0.15	21	61.9 (13)	0.051	0.19
Mood (affective) disorders	15	26.7 (4)	0.745	0.03	1	100.0 (1)	0.431	0.11
Disorders of psychological development	5	0	n/a	n/a	5	20.0 (1)	0.387	− 0.10
Other disorders	2	50 (1)	0.415	0.09	2	0	n/a	n/a

Chi-square test. Fisher’s exact is applied when n < 10. Significant associations are marked with boldface

*ES* effect size, calculated as Cramer’s V

**Table 5:** Logistic regression analysis of associations between demographic characteristics, maltreatment type, mental disorders and recommendations for child and adolescent mental health services (CAMHS)

	Ages 0–6 (*n* = 94)			Ages 7–17 (*n* = 100)		
	OR	95% CI	*p*	OR	95% CI	*p*
Gender						
Boys	1.85	0.46–7.40	0.385	1.27	0.52–3.08	3.079
Age						
Per 1-year increase	1.31	0.88–1.95	0.091	**1.20**	**1.03–1.41**	**0.022**
Maltreatment type						
Physical or sexual abuse	0.80	0.18–3.58	0.775	1.10	0.44–2.71	0.844
Mental disorder						
Neurotic stress-related and somatoform disorders	**23.6**	**5.65**–**98.79**	**< 0.001**	1.30	0.55–3.10	0.552
Behavioral and emotional disorders	2.68	0.58–12.35	0.207	2.64	0.93–7.54	0.069
Mood disorders	*	*		*	*	
Disorders of psychological development	*	*		*	*	
Other disorders	*	*		*	*	

Significant associations are marked with boldface

### Associations Between Service Need in General and Age, Gender, Maltreatment Type, and Mental Disorders

A standard multiple regression was performed to test the influence of age, gender, maltreatment type and mental on the need for services in general (Table 6). For children aged 0–6, male gender (*b* = 0.99, *p* = .007) and stress-related and adjustment disorders (*b* = 1.09, *p* = .018) were significantly associated with the number of recommended services (*R*^*2*^ full model = 0.24). For children in the older age group, having a behavioral and/or emotional disorder was significantly associated with the number of recommended services (*b* = 1.05, *p* = .008; *R*^*2*^ full model = 0.09). Age (within each age cohort) and exposure to sexual or physical abuse was not associated with service need in general.

**Table 6:** Multiple regression analysis of associations between age, gender, maltreatment type, mental disorders and the number of services recommended

	Ages 0–6 (n = 94)				Ages 7–17 (n = 100)			
	*b*	*p*	95% CI	*R*^*2*^	*b*	*p*	95% CI	*R*^*2*^
				**0.24**				**0.09**
Gender								
Boys	**0.99**	**0.007**	**0.28–1.70**		− 0.21	0.516	− 0.87–0.44	
Age								
Per 1-year increase	0.16	0.104	− 0.34–0.36		− 0.02	0.793	− 0.13–0.10	
Maltreatment type								
Physical or sexual abuse	0.08	0.842	− 0.70–0.86		− 0.05	0.888	− 0.71–0.62	
Mental disorder								
Neurotic, stress-related and somatoform disorders	**1.09**	**0.018**	**0.19–1.20**		0.03	0.910	− 0.59–0.66	
Behavioral and emotional disorders	− 0.10	0.830	− 1.05–0.85		**1.05**	**0.008**	**0.29–1.83**	
Mood disorders	− 0.80	0.116	− 1.80–0.20		1.09	0.495	− 2.08–4.26	
Disorders of psychological development	0.35	0.662	− 1.23–1.93		− 0.15	0.825	0.28–1.83	
Other	1.68	0.167	− 0.71–4.07		− 1.09	0.324	− 3.28–1.10	

Significant associations are marked with boldface

## Discussion

This study is part of a larger evaluation of a comprehensive assessment model for children in OOHC in Norway. Through the CARE assessment model, extensive needs across a range of services were identified. The clinicians recommended services outside of the CWS for 92% of the children. Primary health care services and EPS were most frequently recommended. As expected, the prevalence of mental disorders was high, both among younger (50%) and older children (69.6%). Most children with mental disorders were not recommended referral to CAMHS, as there was a tendency to wait and see before such recommendations. This emphasizes the need for continued monitoring of needs over time for these children. The majority of service needs were previously undetected, which illustrate the importance of offering comprehensive assessment routinely to children in OOHC. The amount of service needs documented also calls for close inter-agency collaboration, and has implications for the planning and dimensioning of services. Finally, further evaluation of assessment models is warranted to meet the needs of this high-risk population.

### Identification of Needs Through Comprehensive Assessment

Our results are consistent with previous studies, identifying high levels of service needs, and extensive mental health difficulties among children in OOHC. Only a tiny minority of the children in our study were already in contact with services at the time of the assessment, illustrating the high number of undetected and unmet needs in this high-risk child population. The majority of the children assessed were in need of multiple services, in addition to the CWS. In comparison, a recent Norwegian study of service use among adolescents in foster care, reported that only one third were in contact with two services or more outside the CWS [36]. The comprehensiveness of the CARE assessment model presented here, leads to extensive information about the child and its need for a wide range of health and educational services, as well as need for individualized care. Allocating resources to such a comprehensive assessment may be challenging [2]. However, knowing the rates of the (un)identified needs, and the impact of unmet needs, reducing the scope of the assessment is hardly justifiable. The category of watchful waiting, frequently used by the teams, may reflect an expectation for improvement in a stable care situation. For this to be useful, close monitoring and/or reassessment of the child’s health and development is required. A reassessment may also function as a follow-up, ensuring that the services offered to the child and its career lead to the expected outcomes.

### Mental Disorders and Service Needs

The prevalence of mental disorders found in our study, are higher than reported in prevalence studies of children in the CWS [10], but in accordance with prevalence rates identified through similar comprehensive health assessments, using both clinical interviews and standardized measures [2, 12]. It is surprising that new or continued contact with CAMHS was recommended for less than 50% of children with an identified mental disorder. High-quality care, the supervision of carers according to the specific needs of the child, and community health care may be seen as the first line treatment for disturbed attachment and exposure to abuse and/or neglect in the birth families. The presence of stress-related and adjustment disorders was associated with recommended referral to CAMHS for younger children. For older children, of which nearly two thirds were diagnosed with with PTSD or other reactions to severe stress, the presence of these disorders were not associated with referral to CAMHS. This may indicate a higher threshold for referring older children with this symptomatology to CAMHS than their younger peers. Clinicians may have had expectations for symptom reduction in the new care situation, and saw other services as sufficient. There may be a similar rationale behind the recommendation that was made for 42% of children in the older age cohort, to “watchfully wait” before referring them to CAMHS. Our results imply that traditional approaches for assessment and treatment in CAMHS may not be considered helpful for the complex symptomatology these children present with. A meta-analysis of evidence-based psychotherapies for youth reported that the therapies did not have the same effect on patients with complex symptom patterns or high comorbidity [57].

Specific mental disorders were associated with the need of a higher number of services. This pattern of associations between high levels of mental health difficulties and overall service need are in accordance with findings from studies of service use among children in foster care, from Scotland and Norway [13, 36].

### Maltreatment Type and Service Needs

Exposure to physical and sexual abuse was associated with recommendations for CAMHS among younger children in the bivariate analyses, whereas the adjusted regression model showed no association between the two. CWS registration and classification of maltreatment may be too simplistic, and may not reflect the true impact of the maltreatment, as has been suggested in previous findings [58]. To more accurately measure the effect of maltreatment, information concerning the severity, duration, age of onset, and frequency of the maltreatment needs to be included. This however, was beyond the scope of this study, and is often beyond what the CWS have access to as a child enters OOHC.

A potential explanation for the association no longer being significant could be related to over-adjustment bias [59]. If exposure to maltreatment and these specific disorders are causally linked, and placed in the same model, there is a chance for “masking” the true effect of physical and sexual abuse on need for CAMHS.

### Age, Gender and Service Needs

The difference in the mean number of services recommended between the two age groups may reflect that entry into care at a younger age is beneficial. Further, reaching some developmental milestones can only be determined at the expected age, which may result in a wait-and-see attitude towards developmental problems in the younger age group. The association between increasing age and greater need for mental health services in the older age group may be an effect of older age at entry into care, which is in accordance with previous studies [47]. Special attention should be paid to the service needs of the youngest children as they have been found to receive less services than older preschoolers, even with the same risk levels [60]. The effect of gender on service needs found in the younger age group supports previous findings of boys needing more health care services than girls [38, 49]. Gender did not influence overall service need among the older children.

### Implications for Policy and Practice

The service needs described in our results are substantial and warrant serious attention from the CWS, the health care services, educational services, and policymakers. In the absence of a thorough health assessment, it is likely that this high-risk population goes un- and underserved, with detrimental effects on health and adjustment to independent adult life. Our results support previous studies emphasizing the importance of specialized clinicians using a trauma-informed, systematic approach to assess the mental, physical health and developmental needs of children living in OOHC [48]. Comprehensive, early assessment provided to all children after placement, is crucial to identify needs, plan treatment and provide foster carers with the information and supervision necessary to support their child. Children in OOHC poses a challenge to the services involved in their care, and understanding the extent to which specific services are needed may contribute to the planning and dimensioning of services. The vast majority of children assessed in our study were in need of a number of external services, in addition to foster care and support from the CWS. This finding emphasizes the necessity of developing a comprehensive health plan and systems for interagency collaboration. Furthermore, our results imply that there is tendency to “wait and see” with regards to referral to specialized mental health services, even when criteria for mental disorders are met, emphasizing the importance of monitoring and reassessing health care needs, specifically concerning their mental health.

Identifying needs and recommending referrals are only the first steps on the way to improving health and development for these children. There is a need for a system with clearly assigned responsibility for coordinating and monitoring the delivery of services and facilitating collaboration between services, in order to avoid fragmentation and diffusion of responsibility. Providing appropriate support for foster carers is critical, both because of the potential for repair that lies within the relationship between child and carer [12], and because they are the ones who navigate through the services with the children [48]. Finally, the complex difficulties characteristic for children in OOHC, may not be treated through the traditional approaches offered by CAMHS. In accordance with previous studies our results imply that there is a need for evaluating current CAMHS practices for service provision to this child population as the traditional approaches do not seem to target their needs [13, 48].

### Strengths and Limitations

The major strength of our study is the advantage of the assessment reports stemming from a comprehensive diagnostic examination conducted by a multidisciplinary team of dedicated clinicians using standardized methods. Seeing the child over the course of several hours and collecting information from multiple informants, adds to the reliability and credibility of the assessment results. The majority of children took part in the assessment around 6 months after placement, a time when the most immediate reactions to placement and to the CWS investigation may have subsided, thus reflecting more stable needs of the child.

However, the results of this study should be interpreted within the context of some limitations. Stronger conclusions and increased generalizability may have been possible if all children placed in OOHC from this region had been referred for the assessment shortly after placement. Referral rates differed between CWS offices, as did the time between placement and referral to assessment. We did not have full access to information about ethnicity, social background and the health of birth parents thus potentially important confounding variables could not be accounted for. A further limitation concerns the age breakdown in our analyses, due to the involvement of two different teams assessing different age cohorts. This makes it difficult to disentangle the effect of age from the effect of assessment team when comparing the two age groups. A larger sample size would have given the study greater statistical power to explore associations, but given the resources available in this pilot study, this was not possible. As no formal power calculations were carried out, the results should be treated as preliminary and interpreted with caution.

Furthermore, when variables that may be causally related are included in the same regression model, there is a risk for overadjustment bias. Finally, cross sectional data gathered at one point may yield primarily situational results versus more stable characteristics and limits the ability to draw causal inferences.

### Summary

Exposure to child maltreatment and other risk factors make children in OOHC a particularly vulnerable population with extensive physical, mental and developmental needs. International guidelines recommend comprehensive health assessments for all children in OOHC. The present study sought to describe the mental disorders and the recommended services identified through a multidisciplinary comprehensive health assessment model, and to explore the influence of explanatory factors on the need for mental health services and for services in general.

Our results show the need for a broad range of services, with 39% of children aged 0 to 6 years and 71% of children aged 7 to 17 years needing support from three services or more, outside the CWS. Only 8% of the children had no need for additional services. Mental disorders were identified in 50% of children aged 0–6 years, and in 70% of children aged 7–17 years. The presence of stress-related and adjustment disorders in younger children and emotional and behavioral disorders in older children, were positively associated with overall service need. However, less than half of the children with a mental disorder were recommended referral to CAMHS. Recommendations for CAMHS was associated with male gender and stress-related disorders for younger children, and with increasing age for older children. Our results suggest that the CARE assessment model was effective in identifying children with unmet needs that may otherwise have missed detection. Our findings justify routine, trauma-informed assessment of all children entering care and suggest that the assessment may need to be an ongoing process. The broad range of recommended services and the high prevalence of mental disorders identified in our study, emphasize the importance of conducting a comprehensive multi-disciplinary assessment, and necessitates coordination of service delivery and inter-agency collaboration. Our findings also imply the need for further development of population-specific interventions.

There is limited knowledge of the effect of assessment when it comes to service provision and the long-term health improvements for the children. There is a need for further studies to examine if the children are referred to and receive the recommended services, and if the services are effective in meeting the individual needs of the children. Future research should also explore how the recommendations for foster carer supervision are followed through by the CWS and how the foster carers experience the recommended supervision. A follow-up of the current sample has been implemented, examining referral rate after assessment, child service use and child mental health 12 months after the assessment.

## Supplementary Information

Below is the link to the electronic supplementary material.

Supplementary material 1 (DOCX 16.2 kb)

## Data Availability

The data required to reproduce the above findings cannot be shared at this time due to ethical reasons.
