# Supplementary material for: Comprehensive Health Assessment for Children in Out-of-Home Care: An Exploratory Study of Service Needs and Mental Health in a Norwegian Population
Source: Child Psychiatry Hum Dev. 2023 Oct 12;56(4):958–70. doi: 10.1007/s10578-023-01619-5 (PMC12289769; doi:10.1007/s10578-023-01619-5)
Supplement: Supplementary file 1 — Supplementary material 1 (DOCX 16.2 kb) [file 10578_2023_1619_MOESM1_ESM.docx]

**Table S1:** Measures included in the CARE assessment model for children entering OOHC

| **Measures** | **Description** | **Pre-assessment** | **Assessment**  **day** |
| --- | --- | --- | --- |
|  |  |  |  |
| **Psychicatric diagnosis** |  |  |  |
| The Development and Well-Being Assessment (DAWBA ) | Interviews, questionnaire and rating techiniques to assess psychiatric diagnoses in children with children, parents and teachers as informants, ages 2-17 years | X | X |
|  |  |  |  |
| **Trauma symptoms** |  |  |  |
| Child and Adolescent Trauma Screen (CATS) | Screening questionnaire for post-traumatic stress symptoms | X | X |
| Trauma Symptom Checklist for Young Children (TSCYC) | Standardized broad trauma measure for young children, ages 3-12 years |  | X |
| Trauma symptom Checklist for Children (TSCC) | Standardized broad trauma measure for children, ages 10-17 years. |  | X |
| Checklist for psychosocial stressors / potentially traumatic events | Checklist based on the diagnostic classification systems DC: 0-5 and ICD-10. |  | X |
|  |  |  |  |
| **Attachment and parent child relationship** |  |  |  |
| Reactive Attachment and Disinhibited Social Engagement Disorder Assessment (RADA) | Semi structured interview for assessing the symptoms of Reactive attachment disorder and disinhibited social engagement disorder |  | X |
| The Crowell procedure | An assessment tool used to assess parent-child relationship (adapted for the current purpose) |  | X |
| **Sensory functioning** |  |  |  |
| Test of Sensory Functioning in Infants (TSFI) | Test to identify infants with sensory integrative dysfunction, for children from 4 to 18 months |  | X |
|  |  |  |  |
| **Executive functions** |  |  |  |
| Behavior Rating Inventory of Executive Functioning (BRIEF) | Executive functions in children, ages 5-18 years |  | X |
|  |  |  |  |
| **Cognitive ability** |  |  |  |
| Wechsler Preschool And Primary Scale of Intelligence (WPPSI-IV) | Test for measuring cognitive ability, 2-7 years. |  | X |
| Wechsler Intelligence Scale for Children, fourth edition (WISC-IV) | Test for measuring cognitive ability, ages 6-16 years. |  | X |
|  |  |  |  |
| **Additional measures** |  |  |  |
| Alarm Distress Baby Scale (ADBS) | Measures social withdrawal in infants, ages 0-2 years |  | X |
| Toronto Alexithymia Scale (TAS – 29) | Self-administered questionnaire that measures difficulties with describing and identifying feelings, ages 16+ |  | X |
| Screen for violence risk (V-RISK-10) | 10-item brief screen for violence risk, ages 16+ |  | X |
